# Supplementary material for: Predicting age from binarized human oral microbial data combined with an ensemble of classifiers
Source: mSystems. 2025 Oct 31;10(11):e01182-25. doi: 10.1128/msystems.01182-25 (PMC12625745; doi:10.1128/msystems.01182-25)
Supplement: Supplemental Material — Fig. S1 and S2 and supplemental table captions. [file msystems.01182-25-s0001.docx]

**Predicting age from binarized human oral microbial data combined with an ensemble of classifiers**

**Supplementary material**

**Supplementary Tables (Table S1 - 10) ................................................2**

**Supplementary Figures (Fig. S1 - 2) ...................................................4**

**Table S1-1** The relative abundance of phyla in this study.

**Table S1-2** The mean values of phyla in three age groups.

**Table S1-3** The Kruskal-Wallis test and Dunn's post hoc test at the phylum level.

**Table S1-4** The Kruskal-Wallis test and Dunn's post hoc test at the genus level.

**Table S2** The LEfSe analysis result of all taxa levels.

**Table S3-1** Signal-to-noise ratios (SNR) of individual ASVs under four data processing methods.

**Table S3-2** Statistical comparison of SNR values between different data processing methods using Wilcoxon signed-rank test.

**Table S3-3** Pairwise comparisons of SNR values between different data processing methods.

**Table S3-4** Number of ASVs with SNR greater than 1.

**Table S4-1** The 89 ASVs selected from the training set (n = 135) for constructing the ensemble model using binarized oral microbial data.

**Table S4-2** The 121 ASVs selected from the training set (n = 135) for constructing the ensemble model using CLR-transformed data.

**Table S4-3** The 118 ASVs selected from the training set (n = 135) for constructing the ensemble model using log2-transformed data.

**Table S4-4** The 26 ASVs selected from the training set (n = 135) for constructing the ensemble model using relative abundance data.

**Table S5-1** Summary of hyperparameter tuning for the ensemble model across four data processing methods.

**Table S5-2** Summary of hyperparameter tuning for the traditional model across four data processing methods.

**Table S6** The predictive results of eight machine learning algorithms and four data processing methods across age bins with widths ranging from 1 to 64 years.

**Table S7** The prediction results under different age ranges on independent validation set.

**Table S8-1** Five-fold cross-validation results of the ensemble model and Huang's model (n = 2550).

**Table S8-2** Statistical comparison between two models using Wilcoxon signed-rank test.

**Table S9-1** Cross-sex age prediction performance using sex-stratified training and testing sets.

**Table S9-2** Age prediction performance with and without sex as a dummy variable.

**Table S9-3** Comparison of age prediction performance with or without inclusion of sex as a dummy variable using Wilcoxon signed-rank test.

**Table S10-1** Distribution of 150 samples between training and testing sets.

**Table S10-2** Distribution of 2550 samples between training and testing sets.


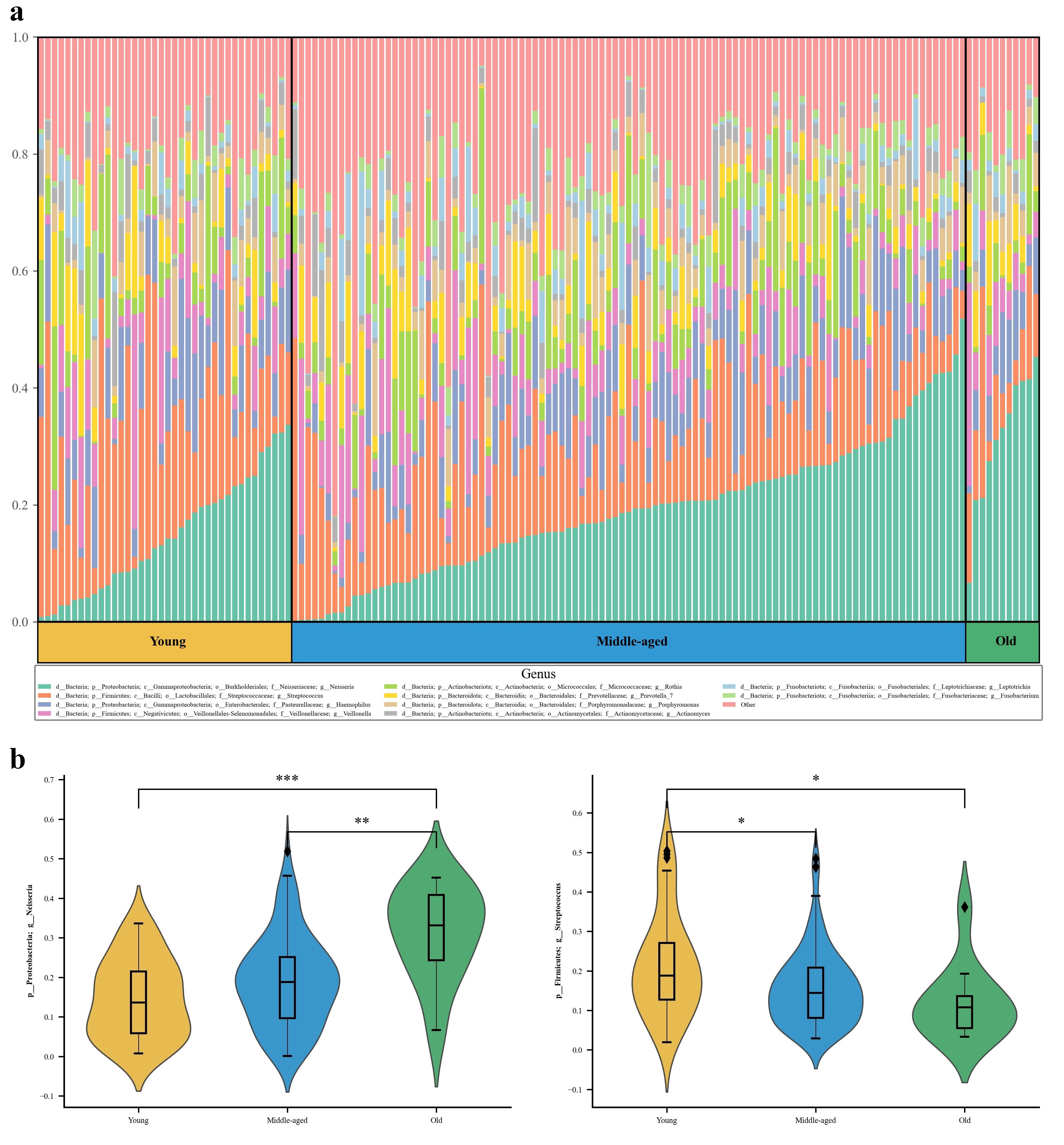


**Fig. S1** (a) Relative abundance of oral bacteria grouped by age at the genus level. (b) Violin plots of genera exhibited statistically significant differences (p value of Kruskal-Wallis test < 0.05) across the three age groups (p value was calculated using the Kruskal-Wallis test followed by Dunn's post hoc test). Young (0-29 years), middle-aged (30-64 years), and old (≥ 65 years). *p < 0.05, **p < 0.01, ***p < 0.001.


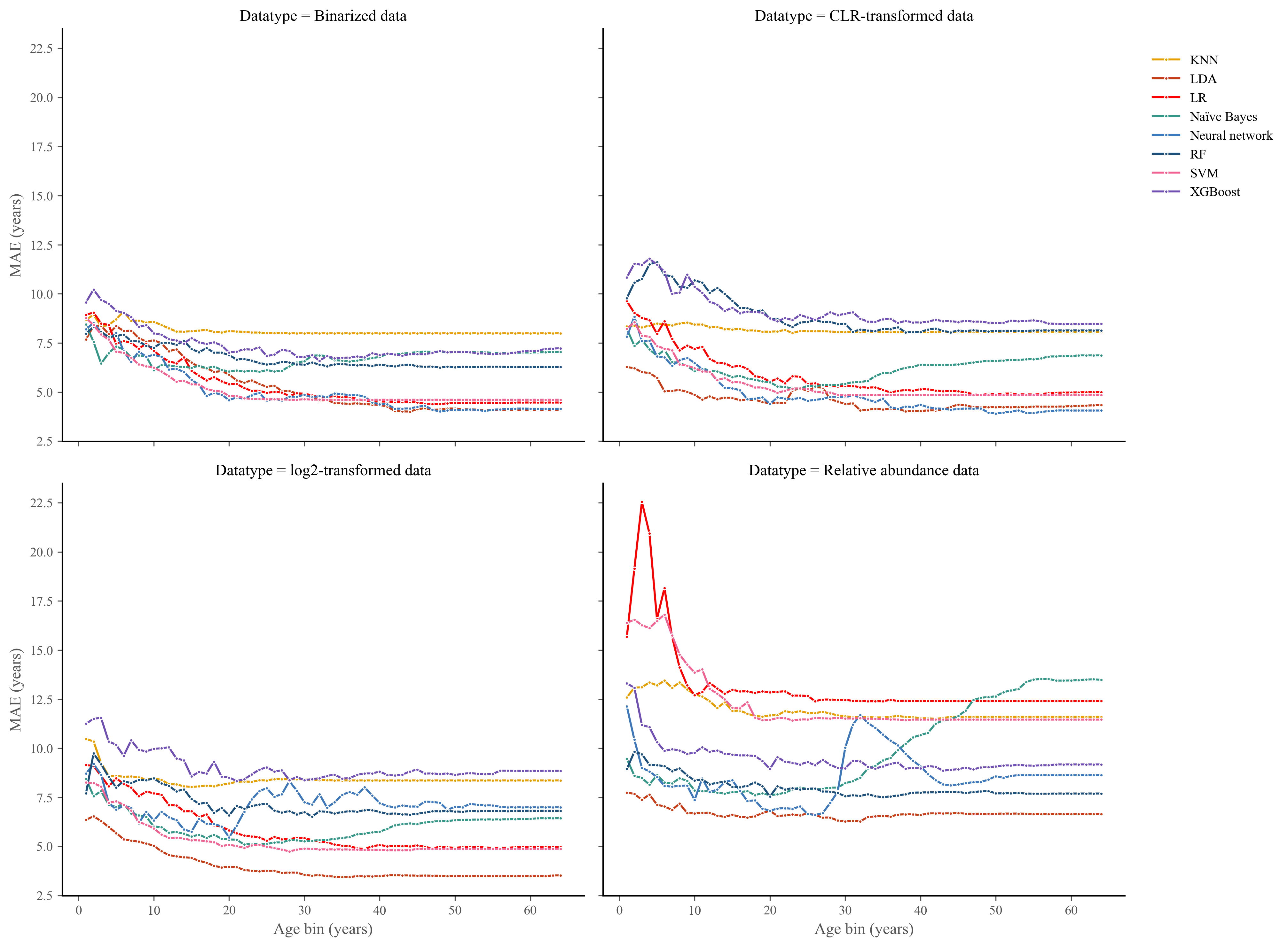


**Fig. S2** Comparison of training set MAE across eight ensemble models for age prediction using four microbial data processing methods across varying age bin widths (1–64 years).
